# The SUMOylation of TAB2 mediated by TRIM60 inhibits MAPK/NF-κB activation and the innate immune response

**DOI:** 10.1038/s41423-020-00564-w

**Published:** 2020-11-12

**Authors:** Zhiwen Gu, Xueying Chen, Wenyong Yang, Yu Qi, Hui Yu, Xiaomeng Wang, Yanqiu Gong, Qianqian Chen, Bo Zhong, Lunzhi Dai, Shiqian Qi, Zhiqiang Zhang, Huiyuan Zhang, Hongbo Hu

**Affiliations:** 1 Department of Rheumatology and Immunology, National Clinical Research Center for Geriatrics, State Key Laboratory of Biotherapy, West China Hospital, Sichuan University, and Collaborative Innovation Center of Biotherapy, Chengdu, 610041 China; 2 Department of Virology, College of Life Sciences, Department of Immunology, Medical Research Institute, Wuhan University, Wuhan, 430072 China; 3 Department of General Practice and Lab of PTM, State Key Laboratory of Biotherapy, West China Hospital, Sichuan University, and Collaborative Innovation Center of Biotherapy, Chengdu, 610041 China; 4 Department of Urology, State Key Laboratory of Biotherapy, West China Hospital, Sichuan University, and Collaborative Innovation Center of Biotherapy, Chengdu, 610041 China; 5 Immunobiology and Transplant Science Center, Houston Methodist Hospital, Houston, TX USA 77030

**Keywords:** SUMOylation, innate immune response, TRAM60, TAB2, Innate immunity, Cell signalling

## Abstract

Activation of the TAK1 signalosome is crucial for mediating the innate immune response to pathogen invasion and is regulated by multiple layers of posttranslational modifications, including ubiquitination, SUMOylation, and phosphorylation; however, the underlying molecular mechanism is not fully understood. In this study, TRIM60 negatively regulated the formation and activation of the TAK1 signalosome. Deficiency of TRIM60 in macrophages led to enhanced MAPK and NF-κB activation, accompanied by elevated levels of proinflammatory cytokines but not IFN-I. Immunoprecipitation-mass spectrometry assays identified TAB2 as the target of TRIM60 for SUMOylation rather than ubiquitination, resulting in impaired formation of the TRAF6/TAB2/TAK1 complex and downstream MAPK and NF-κB pathways. The SUMOylation sites of TAB2 mediated by TRIM60 were identified as K329 and K562; substitution of these lysines with arginines abolished the SUMOylation of TAB2. In vivo experiments showed that TRIM60-deficient mice showed an elevated immune response to LPS-induced septic shock and *L. monocytogenes* infection. Our data reveal that SUMOylation of TAB2 mediated by TRIM60 is a novel mechanism for regulating the innate immune response, potentially paving the way for a new strategy to control antibacterial immune responses.

## Introduction

Innate immune cells recognize and respond to various pathogens through their pattern recognition receptors (PRRs). When conserved pathogen-associated molecular pattern (PAMP) molecules bind to cognitive PRRs, signaling cascades are activated for the transcription of genes encoding proinflammatory cytokines and type I interferon (IFN-I).^1^ Among PRR pathways, Toll-like receptor (TLR) signaling pathways have been intensively studied. The myeloid differentiation primary response gene 88 (MyD88)-dependent pathway is activated in all TLR-mediated signaling pathways except for TLR3.^2^ The critical step in this pathway is to activate TGF-β-activated kinase 1 (TAK1), which requires ubiquitination mediated by TNF receptor-associated factor 6 (TRAF6).^3^ Upon stimulation, TRAF6 is recruited to IL-1 receptor-associated kinase 1 (IRAK1) together with the E2 ubiquitin-conjugating enzyme UBC13, resulting in the K63-ubiquitination of IRAK1 and autoubiquitination of TRAF6.^4^ TRAF6-conjugated K63-ubiquitin chains serve as scaffolds to recruit the TAK1/TAK1 binding protein 2 (TAB2)/TAB3 complex through the Npl4 zinc-finger (NZF) domain of TAB2, which activates TAK1 and downstream MAPK and NF-κB signaling pathways.^5,6^ TRAF6/UBC13 also catalyzes the synthesis of free polyubiquitin chains that are reported to interact with TAB2 and activate TAK1.^7^ TAK1 is activated by a similar mechanism in TNF receptor-induced MAPK and NF-κB pathways. In this case, TRAF2 and cellular inhibitor of apoptosis protein (cIAP) function as E3 ligases to mediate the K63-ubiquitination of receptor-interacting protein 1 (RIP1), which in turn recruits the TAK1 complex via the binding of TAB2/TAB3 to K63-ubiquitin chains.^8^ Despite the requirement of TAK1, the roles of TAB2 and TAB3 in these pathways are debatable, as argued by studies using TAB2/TAB3-deficient mouse embryonic fibroblasts (MEFs).^9–11^ Additional studies also suggest that the function of TAB2 in MAPK and NF-κB activation is cell type- and/or stimulus-specific.^11,12^ Nevertheless, TAB2 expression is targeted by ubiquitin E3 ligases^13–15^ and miRNAs^16,17^ to modulate the activation of MAPK and NF-κB signaling pathways in macrophages and dendritic cells of both humans and mice. The precise roles of TABs and additional regulatory mechanisms remain illusive.

SUMOylation is a posttranslational modification of proteins that involves the conjugation of small ubiquitin-like modifiers (SUMOs) to lysine residues of target proteins.^18^ In a pattern analogous to that of ubiquitination, this process also requires E1 SUMO-activating enzymes (SAE1 and SAE2), an E2 SUMO-conjugating enzyme (Ubc9) and an E3 SUMO ligase.^19^ A growing number of E3 ubiquitin ligases have been identified as E3 SUMO ligases, including tripartite motif-containing protein (TRIM).^20–22^ The TRIM protein family is composed of over 70 members, which play irredundant roles in the innate immune response to various types of pathogens.^23,24^ These proteins have conserved tripartite motifs, i.e., RING, BBox, and CC domains in the N-termini, and diverse C-termini. In addition to serving as E3 ubiquitin ligases, TRIMs could also act as E3 SUMO ligases, as indicated by a growing body of studies. TRIM19, also known as promyelocytic leukemia protein (PML), promotes SUMOylation and stabilization of p53.^25,26^ Similarly, TRIM27 mediates the SUMOylation of MDM2, resulting in the stabilization of MDM2.^25^ TRIM38 is reported to mediate the ubiquitination and degradation of TRAF6 and nucleosome assembly protein 1 (NAP1), suppressing the activation of downstream NF-κB and interferon regulatory factor 3 (IRF3).^27,28^ TRIM38 also acts as the SUMO ligase for melanoma differentiation-associated protein 5 (MDA5) and retinoic acid-inducible gene-I (RIG-I), which suppresses the K48-ubiquitination and degradation of these proteins, thus promoting the host antiviral innate immune response to RNA viruses.^29^ However, whether and how TRIM-mediated SUMOylation regulates TLR signaling and the underlying mechanisms need to be further investigated.

Comprehensive screening of all human TRIM proteins indicates the potential of TRIM60 in the NF-κB pathway and innate immune response,^30^ but how TRIM60 modulates the innate immune response is unknown. In this study, our data indicated that TRIM60 served as a negative regulator of TLR4/TLR9 signaling pathways. In TRIM60-deficient macrophages, MAPK and NF-κB activation were enhanced, leading to elevated proinflammatory cytokine production upon stimulation by lipopolysaccharide (LPS) and CpG-ODN1826 (referred to as CpG hereafter). A mechanistic study indicated that TAB2 was the TRIM60-interacting protein and targeted for SUMOylation. The SUMOylation of TAB2 suppressed the formation of the TRAF6/TAB2/TAK1 complex and downstream signals. In vivo studies suggested that TRIM60-deficient mice were more susceptible to LPS-induced septic shock but more resistant to *Listeria monocytogenes* infection than control mice. Taken together, our data reveal a novel posttranslational modification of TAB2, SUMOylation mediated by TRIM60, as a negative regulatory mechanism of the innate immune response via TLR signaling to avoid tissue damage caused by an overwhelming immune response.

## Results

### TRIM60 suppresses proinflammatory cytokine production in macrophages

**Fig. 1:**
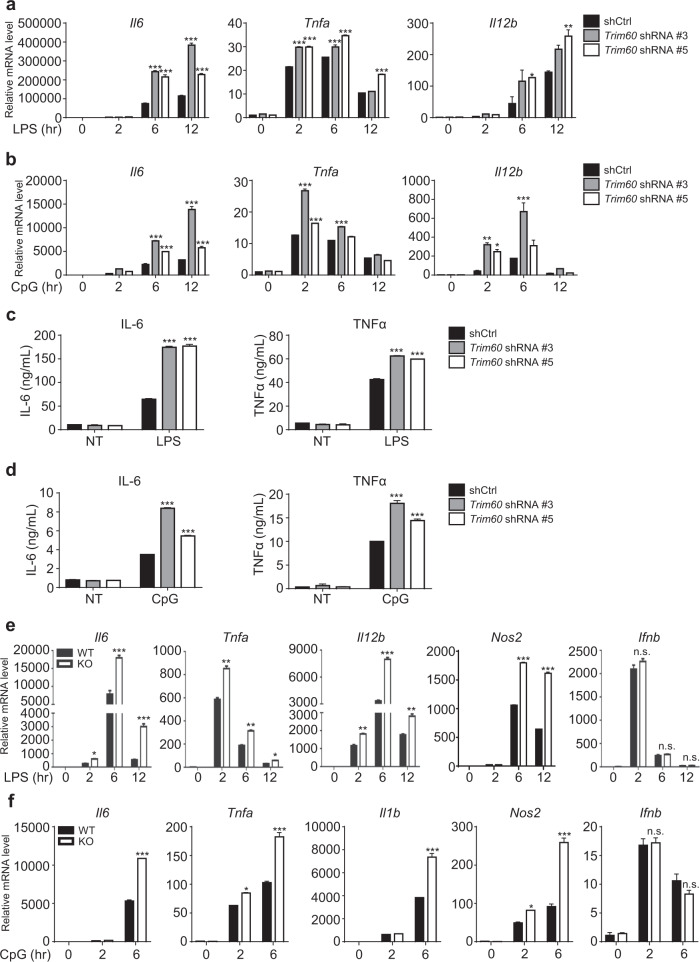
TRIM60 negatively regulates TLR-mediated proinflammatory cytokine production. qPCR analysis of *Il6*, *Tnfa*, and *Il12b* expression in control (shCtrl) and *Trim60* shRNA-expressing (*Trim60*-shRNA #3, sh3; *Trim60*-shRNA #5, sh5) RAW cells stimulated with either LPS (**a**) or CpG (**b**) for the indicated amounts of time. **c, d** ELISA analysis of proinflammatory cytokine production in control and TRIM60-knockdown RAW cells stimulated with LPS or CpG. The indicated cells were left untreated or stimulated with either LPS (**c**) or CpG (**d**) for 12 h before ELISA analysis of IL-6 and TNFα production. **e, f** qPCR analysis of proinflammatory cytokine expression in BMDMs. Wild-type (WT) and TRIM60-deficient BMDMs were stimulated with either LPS (**e**) or CpG (**f**) for the indicated time periods and then subjected to qPCR analysis of proinflammatory cytokine expression. The data in **a, b, e,** and **f** are presented relative to the *18S rRNA* expression level. The data in **a–f** are shown as the mean ± SEM and are representative of at least three independent experiments. *P < 0.05; **P < 0.01; ***P < 0.001; n.s. no significance (two-way ANOVA followed by Tukey’s multiple comparisons)

Negative feedback mechanisms have been developed by hosts to maintain immune homeostasis.^31^ We found that the mRNA level of *Trim60* was downregulated upon LPS and CpG stimulation (Supplementary Fig. 1), suggesting that TRIM60 might play a crucial role in TLR signaling pathways. To examine this hypothesis, we employed shRNA to knock down *Trim60* expression in the macrophage cell line RAW 264.7 (referred to as RAW cells hereafter). Two *Trim60*-specific shRNAs and one control shRNA were introduced into RAW cells via lentiviral vectors. The knockdown efficiency was examined by real-time quantitative PCR (qPCR) and western blot (WB). Compared with the control shRNA, both *Trim60*-specific shRNAs significantly reduced the *Trim60* mRNA levels in RAW cells (Supplementary Fig. 2a) and the TRIM60 protein levels in transfected HEK293T cells (Supplementary Fig. 2b). These cells were then stimulated with either LPS or CpG, and proinflammatory cytokine production was measured by qPCR and ELISA. TRIM60 knockdown in RAW cells resulted in significantly enhanced expression of proinflammatory cytokines, including *Il6*, *Tnfa*, and *Il12b*, but not *Ifnb* (Fig. 1a–d; Supplementary Fig. 3a, b), implying that TRIM60 served as a negative regulator in LPS- and CpG-activated MyD88-dependent signaling pathways.

To study the biological function of TRIM60 in vivo, *Trim60-floxed* mice were generated by inserting two *loxp* sites upstream and inside of the second exon of *Trim60* (Supplementary Fig. 4a). *Trim60*^*f/f*^ mice were further crossed with Ella-Cre mice to generate TRIM60-knockout mice. Heterozygous mice (Ella-Cre^+^ *Trim60*^*w/f*^) were bred to generate TRIM60-deficient mice (Ella-Cre^+^ *Trim60*^*f/f*^, referred to as *Trim60*^*−/−*^, KO) and TRIM60-sufficient control littermates (Ella-Cre^+^ *Trim60*^*w/w*^, referred to as wild-type, WT) (Supplementary Fig. 4b, c). qPCR results indicated a deficiency of *Trim60* mRNA in bone marrow-derived macrophages (BMDMs) and MEFs isolated from *Trim60*^*−/−*^ mice (Supplementary Fig. 4d). Although TRIM60 has been reported to be involved in testis development,^32^ germline TRIM60-deficient mice were born at Mendelian ratios with normal growth (data not shown).

To examine the roles of TRIM60 in regulating the innate immune response, we generated BMDMs from TRIM60-deficient mice and control littermate mice and stimulated macrophages with either LPS or CpG. As expected, both stimuli induced proinflammatory cytokine and IFN-I production in WT BMDMs, while they significantly enhanced the expression of cytokines, including *Il6*, *Tnfa*, *Il12b*, and *Nos2*, but not *Ifnb*, in *Trim60*^*−/−*^ BMDMs (Fig. 1e, f). Therefore, our data suggested that TRIM60 specifically regulated TLR-mediated proinflammatory cytokine production but was dispensable for IFN-I production in the tested signaling pathways.

### TRIM60 deficiency potentiates the MAPK and NF-κB signaling pathways

**Fig. 2:**
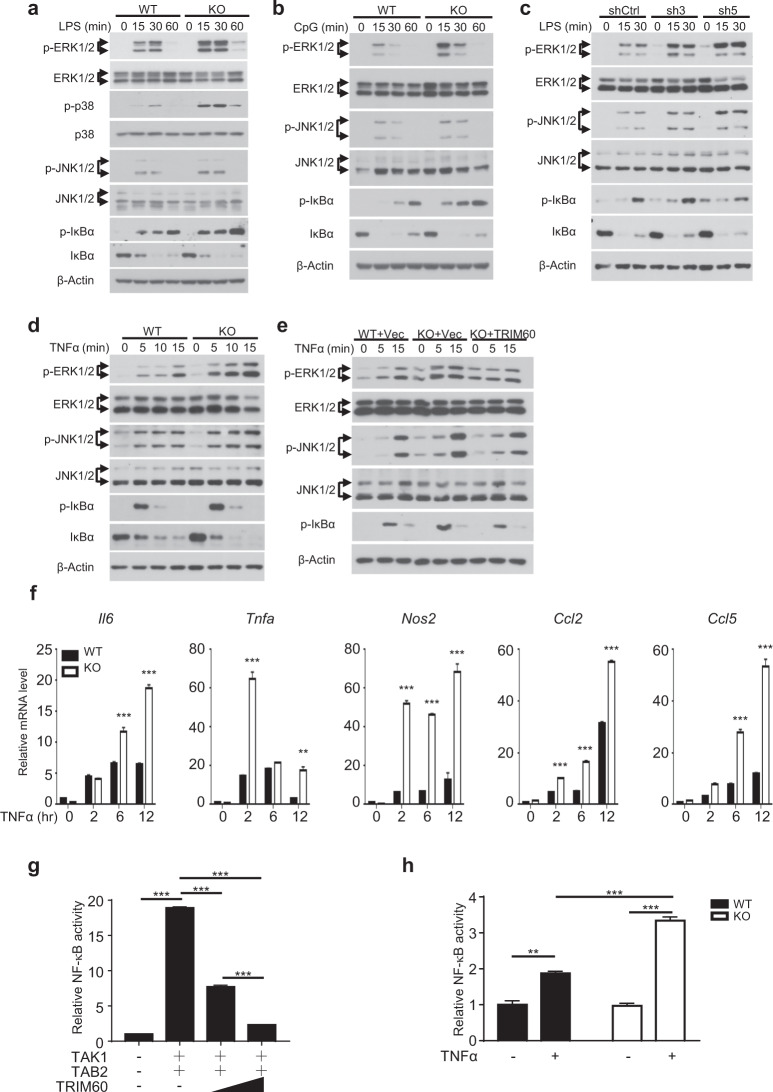
TRIM60 negatively regulates TLR-mediated MAPK and NF-κB signaling activation. **a, b** WB analysis of LPS- or CpG-induced MAPK and NF-κB activation in WT and TRIM60-knockout (KO) BMDMs. BMDMs were untreated or stimulated with either LPS (**a**) or CpG (**b**) for the indicated amounts of time, and WB was then performed to detect the phosphorylation of ERK, p38, JNK, and IκBα. **c** WB analysis of the LPS-induced activation of MAPK and NF-κB in control and TRIM60-knockdown RAW cells. Control (shCtrl) and TRIM60-knockdown RAW cells (sh3 and sh5) were stimulated with LPS as indicated and then subjected to WB to detect ERK, JNK, and IκBα phosphorylation. **d, e** WB analysis of MAPK and NF-κB activation in WT, TRIM60-KO, and TRIM60-reconstituted MEFs stimulated with TNFα. Cells were untreated or stimulated with TNFα for the indicated time periods, and WB was used to detect the phosphorylation of ERK, JNK, and IκBα. For the data in **a–e**, the protein levels of total ERK1/2, p38, JNK1/2 and β-Actin were used as the loading controls. **f** TRIM60 negatively regulates TNFα-induced proinflammatory cytokine and chemokine production. WT and TRIM60-deficient MEFs were stimulated with TNFα as indicated, and *Il6*, *Tnfa*, *Nos2*, *Ccl2*, and *Ccl5* expression was then analyzed by qPCR. The relative gene expression levels were normalized to that of *18S rRNA*. **g** Dual luciferase assay to assess the effects of TRIM60 on TAK1/TAB2-induced NF-κB activity. HEK293T cells were cotransfected with the indicated plasmids together with the NF-κB and *Renilla* control reporter plasmids for 48 h before being subjected to a dual luciferase reporter assay. **h** Dual luciferase assay results indicating the effect of TRIM60 on TNFα-stimulated NF-κB activity. WT and TRIM60 KO MEFs were transfected with the NF-κB reporter plasmid and a *Renilla* control reporter plasmid. Twenty-four hours after transfection, the cells were stimulated with TNFα for 6 h, and the luciferase activity was measured. The firefly luciferase activity levels in **g, h** were normalized to those of *Renilla* luciferase and are presented as the mean ± SEM. **P < 0.01; ***P < 0.001 (one-way ANOVA followed by Tukey’s multiple comparisons for **g**, two-way ANOVA followed by Tukey’s multiple comparisons for **h**). The data are representative of at least three independent experiments (**a–h**)

Both LPS and CpG activated the MyD88-dependent signaling pathway for proinflammatory cytokine production. Our previous data have indicated that TRIM60 plays a critical role in proinflammatory cytokine production in response to both LPS and CpG stimulation, suggesting that TRIM60 might regulate MyD88-dependent MAPK and NF-κB signaling activation. To examine our hypothesis, WT and *Trim60*^*−/−*^ BMDMs were generated and stimulated with LPS or CpG, followed by WB analysis to detect the activation of the MAPK and NF-κB signaling pathways. Upon LPS or CpG stimulation, the phosphorylation of ERK, JNK, and p38, as well as IκBα in *Trim60*^*−/−*^ macrophages was enhanced compared with that in WT cells (Fig. 2a, b). Elevated MAPK and NF-κB activation was also observed in TRIM60-knockdown RAW cells stimulated by LPS (Fig. 2c), indicating that TRIM60 was indeed involved in both the TLR4 and TLR9 signaling pathways.

To explore whether TRIM60 also regulates the MAPK and NF-κB signaling pathways triggered by other stimuli, such as TNFα, we generated MEFs from WT and *Trim60*^*−/−*^ embryos, which were further treated with TNFα. As expected, TNFα stimulation led to the activation of the MAPK and NF-κB signaling pathways, as indicated by the phosphorylation of ERK, JNK, and IκBα. More importantly, the activation of MAPK and NF-κB was notably enhanced in TRIM60-deficient MEFs (Fig. 2d). Consistently, the expression of proinflammatory cytokines and chemokines, including *Il6*, *Tnfa*, *Ccl2*, and *Ccl5*, was also elevated in *Trim60*^*−/−*^ MEFs stimulated by TNFα (Fig. 2f). To further confirm the function of TRIM60 in TNFα-triggered MAPK and NK-κB activation, exogenous TRIM60 was introduced into *Trim60*^*−/−*^ MEFs using lentiviral infection, and the its expression level was determined by WB (Supplementary Fig. 5). In line with the results shown in Fig. 2d, the phosphorylation of ERK, JNK, and IκBα in *Trim60* ^*−/−*^ MEFs infected with the control lentiviral vector was significantly increased compared with that in WT MEFs infected with the control lentiviral vector. Intriguingly, reconstitution of TRIM60 in *Trim60*^*−/−*^ MEFs rescued the hyperactivation of MAPK and NF-κB caused by TRIM60 deficiency (Fig. 2e). These results were further confirmed by the luciferase assay. As shown in Fig. 2g, co-overexpression of TAB2 and TAK1 significantly induced NF-κB activation in HEK293T cells, while cotransfection of TRIM60 led to suppression of NF-κB activation in a dose-dependent manner. To confirm these results, WT and *Trim60*^*−*/*−*^ MEFs were transfected with the NF-κB luciferase reporter plasmid by nucleofection, followed by TNFα stimulation. As shown in Fig. 2h, TNFα stimulation-induced NF-κB activation in MEFs; notably, NF-κB activation was significantly enhanced in *Trim60*^−/−^ MEFs compared with WT MEFs. In summary, these results indicated that TRIM60 might negatively regulate MAPK and NF-κB activation by affecting common compartments in both the TLR and TNFR signaling pathways in multiple types of cells.

### TAB2 is the TRIM60-interacting protein

**Fig. 3:**
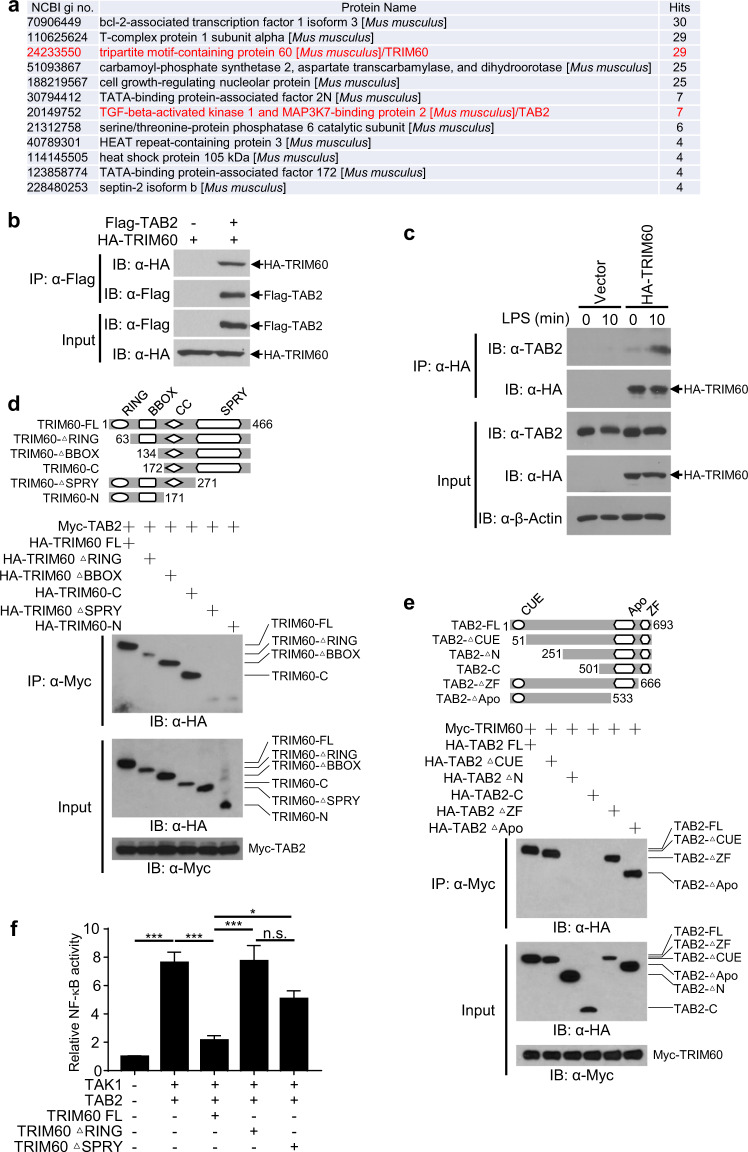
TRIM60 interacts with TAB2 through the C-terminal SPRY domain. **a** Mass spectrometry analysis identified TAB2 as a TRIM60-interacting protein. RAW cells stably expressing HA-TRIM60 were stimulated with LPS for 30 min, followed by IP-MS analysis of TRIM60-interacting proteins. IP and WB analyses were performed to detect the interaction of TRIM60 with TAB2 in the overexpression system (**b**) and in RAW cells (**c**). HEK293T cells transiently transfected with HA-TRIM60 and Flag-TAB2 were subjected to IP and WB (**b**). Control and HA-TRIM60-overexpressing RAW cells were untreated or stimulated with LPS for 10 min, followed by IP and WB assays (**c**). **d**, **e** The IP and WB results showing the critical domains that mediate the TRIM60-TAB2 interaction. HEK293T cells were transiently transfected with the full-length or truncated versions of TRIM60 (**d**) or TAB2 (**e**), followed by IP and WB analyses to determine the domains crucial for the TRIM60-TAB2 interaction. **f** A dual luciferase assay was performed to examine the effects of the RING and SPRY domains of TRIM60 on NF-κB activation. HEK293T cells were cotransfected with the indicated plasmids together with the NF-κB reporter plasmid and a *Renilla* control reporter plasmid. Forty-eight hours after transfection, luciferase activity was measured. The firefly luciferase activity in **f** was normalized to the *Renilla* luciferase activity level and is presented as the mean ± SEM. *P < 0.05; ***P < 0.001; n.s. no significance (one-way ANOVA followed by Tukey’s multiple comparisons). The data are representative of three independent experiments (**b–f**)

To study the molecular mechanism by which TRIM60 regulates the TLR signaling pathway, immunoprecipitation-mass spectrometry (IP-MS) was employed to identify TRIM60-interacting proteins. RAW cells overexpressing HA-TRIM60 were stimulated by LPS. The cells were lysed and subjected to immunoprecipitation with anti-HA magnetic beads, and the immunoprecipitated proteins were detected by mass spectrometry. TAB2, the adapter protein crucial for TAK1 activation, was identified as the TRIM60-interacting protein (Fig. 3a). To confirm the IP-MS results, we overexpressed TRIM60 and TAB2 in HEK293T cells and found that TRIM60 coprecipitated with TAB2, indicating that TRIM60 might form a complex with TAB2 (Fig. 3b). In RAW cells overexpressing HA-TRIM60, the interaction of TRIM60 with TAB2 was also detected (Fig. 3c). Notably, the TRIM60-TAB2 interaction in macrophages was induced by LPS stimulation (Fig. 3c).

To determine the crucial domain(s) mediating the TRIM60-TAB2 interaction, different truncated mutants of TAB2 and TRIM60 were cotransfected into HEK293T cells, followed by immunoprecipitation. The results showed that full-length TRIM60 (FL), TRIM60 ΔRING, TRIM60 ΔBBOX, and TRIM60-C could bind to TAB2, while neither TRIM60 ΔSPRY nor TRIM60-N interacted with TAB2, suggesting that the C-terminus of TRIM60 was responsible for the interaction with TAB2 (Fig. 3d). For TAB2, the N-terminus of TAB2 was required for the TAB2-TRIM60 interaction since neither TAB2 ΔN (defect of amino acid 1-250) nor the C-terminus of TAB2 (TAB2-C) bound to TRIM60. Furthermore, the Apo and zinc-finger (ZF) domains of TAB2 were dispensable for the TAB2-TRIM60 interaction (Fig. 3e). The roles of these critical domains of TRIM60 in NF-κB activation were further studied. In line with the results shown in Fig. 2g, cotransfection of TRIM60 significantly suppressed TAK1/TAB2-induced NF-κB activation. However, TRIM60 without the RING domain (the TRIM catalytic domain generally studied) or SPRY domain (the domain responsible for the TRIM60-TAB2 interaction) failed to inhibit TAB2/TAK1-triggered NF-κB activation (Fig. 3f), indicating that both the RING and SPRY domains of TRIM60 were required for the suppression of TAB2/TAK1-induced NF-κB activation. Collectively, our data suggested that TAB2 was the TRIM60-interacting protein in the regulation of the TLR-mediated signaling pathway.

### TRIM60 mediates TAB2 SUMOylation

Regarding the mechanism by which TRIM60 regulates TAB2, we first examined whether TRIM60 affected the ubiquitination of TAB2. Surprisingly, coexpression of TRIM60 did not induce ubiquitination of TAB2 in HEK293T cells (Supplementary Fig. 6a), and no detectable ubiquitination of TAB2 was detected in RAW cells stimulated with LPS (Supplementary Fig. 6b). Several studies have suggested that TRIM proteins regulate TAB2 degradation in a lysosome-dependent manner;^14,15,33^ however, in our study, there was no appreciable TAB2 degradation upon TLR4 or TNFα stimulation, even for up to 3 h (Supplementary Fig. 6c, d).

**Fig. 4:**
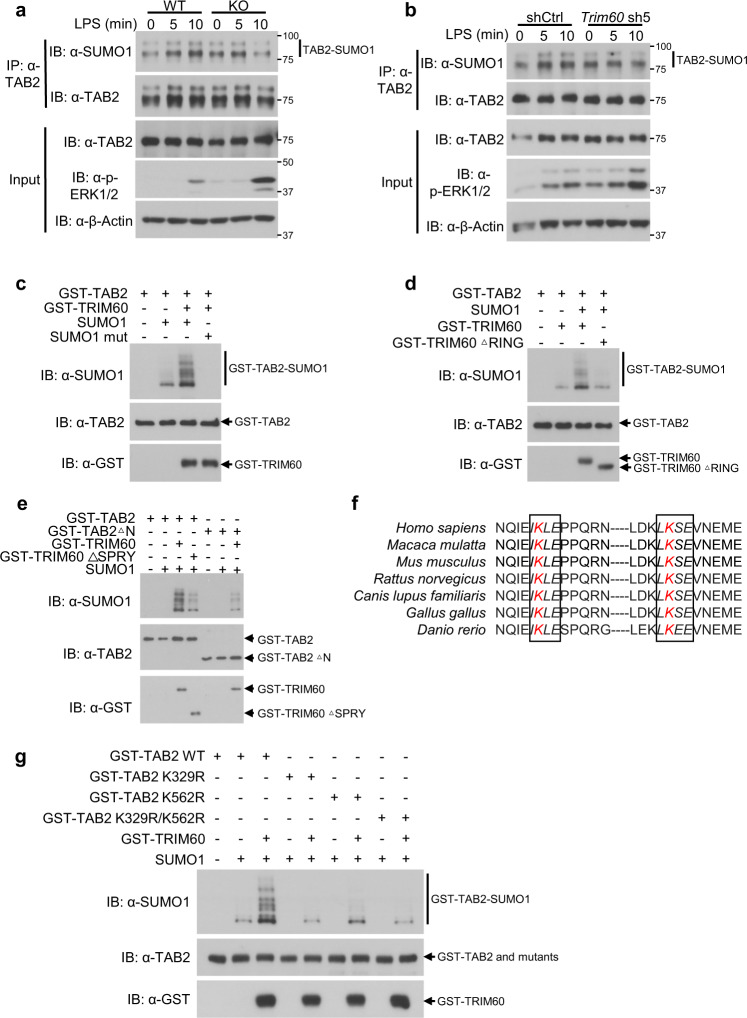
TRIM60 mediates TAB2 SUMOylation. IP and WB analysis of TAB2 SUMOylation. WT and TRIM60 KO BMDMs (**a**) or RAW cells infected with either control shRNA (shCtrl) or *Trim60*-shRNA #5 (*Trim60* sh5) (**b**) were stimulated with LPS for the indicated time durations, followed by IP and WB analyses to examine TAB2 SUMOylation. The relative TAB2 SUMOylation levels (SUMOylated TAB2 vs total TAB2) in **a** and **b** were quantified by ImageJ and are shown as Supplementary Fig. 7a and b, respectively. **c** TRIM60 mediates TAB2 SUMOylation in vitro. GST-TAB2 and GST-TRIM60 were coincubated in vitro in the presence of the E1, E2, and SUMO1 proteins or the mutated SUMO1 protein (SUMO1 mut) at 30 °C for 3h. The samples were analyzed by WB with an anti-SUMO1 antibody. **d** GST-TAB2 was coincubated with either GST-TRIM60 or GST-TRIM60 ΔRING, and the SUMOylation of TAB2 was determined as described in **c**. **e** The TRIM60-TAB2 interaction is required for TAB2 SUMOylation. GST-TAB2, GST-TAB2 ΔN, GST-TRIM60, and GST-TRIM60 ΔSPRY were coincubated as indicated in the in vitro SUMOylation system in the presence of the E1, E2, and SUMO1 proteins, and WB was performed to examine TAB2 SUMOylation. **f** Two consensus SUMOylation motifs (ψ-K-X-E) in TAB2 are conserved among the detected species. The sequences in italics indicate conserved SUMOylation motifs of TAB2. **g** The K329R and K562R mutations abolished TAB2 SUMOylation mediated by TRIM60 as determined by in vitro SUMOylation analysis. The data are representative of three independent experiments (**a**–**e**, **g**)

Given that many E3 ubiquitin ligases also mediate the SUMOylation of target proteins,^25^ it is plausible that TRIM60 might act as an E3 SUMO ligase and target TAB2 for SUMOylation. Whether LPS stimulation could induce the SUMOylation of TAB2 was examined. As shown in Fig. 4a, b, in both primary BMDMs and RAW cells, LPS stimulation induced TAB2 SUMOylation. More importantly, the SUMOylation of TAB2 was compromised in TRIM60-deficient macrophages (Fig. 4a, b and Supplementary Fig. 7a, b), implying that TRIM60 might affect the SUMOylation of TAB2 upon LPS stimulation. An in vitro SUMOylation system using purified proteins was employed to examine whether TRIM60 acts as an E3 ligase to induce the SUMOylation of TAB2. GST-tagged TRIM60 and GST-tagged TAB2 were expressed in *E. coli*, purified, and then incubated with SUMO1 or a SUMO1 mutant in the presence of SUMO E1 enzymes (SAE1/2) and a SUMO E2 enzyme (Ubc9). Incubation of TAB2 in the SUMOylation system without E3 ligase resulted in the basal level of TAB2 SUMOylation, mainly mono-SUMOylation, while TRIM60 robustly enhanced both the mono- and poly-SUMOylation of TAB2 (Fig. 4c). However, the SUMO1 mutant blocked the SUMOylation of TAB2 mediated by TRIM60 (Fig. 4c). The function of the RING domain of TRIM60 in mediating TAB2 SUMOylation was investigated by incubating the GST-TRIM60 ΔRING protein with GST-TAB2 in the SUMOylation system. The results showed that deficiency of the RING domain abolished the capacity of TRIM60 to induce TAB2 SUMOylation (Fig. 4d), which is consistent with the reported catalytic function of the RING domain in TRIMs.^34,35^ To investigate whether the interaction between TRIM60 and TAB2 is required for TAB2 SUMOylation, purified WT TRIM60 or TRIM60ΔSPRY was added to the in vitro SUMOylation system with TAB2 and other components. The results indicated that TRIM60 mediated TAB2 SUMOylation. However, TRIM60ΔSPRY lost the ability to mediate TAB2 SUMOylation (Fig. 4e), which was consistent with the result that the suppression of NF-κB activation by TRIM60 was compromised by deficiency of the SPRY domain (Fig. 3f). Furthermore, TAB2ΔN could not be SUMOylated by TRIM60 (Fig. 4e), suggesting that the SUMOylation of TAB2 also required the N-terminus of TAB2, which mediated the TAB2-TRIM60 interaction (Fig. 3e).

Two lysine residues of TAB2, K329 and K562, were predicted to be SUMOylation sites (sumosp.biocuckoo.org), which is consistent with the canonical consensus motif of ψ-K-X-E.^19^ Moreover, these two lysine residues, as well as these two motifs in TAB2, are highly conserved in the listed species (Fig. 4f) (*Homo sapiens*, *Macaca mulatta*, *Mus musculus*, *Rattus norvegicus*, *Canis lupus familiaris*, *Gallus gallus*, and *Danio rerio*). To study the roles of these lysine residues in TAB2 SUMOylation, the two lysines were substituted with arginines using mutagenesis to generate three TAB2 mutants, TAB2 K329R, TAB2 K562R and TAB2 K329R/K562R. In vitro SUMOylation assays suggested that either a single residue or double residues mutation substantially interrupted the TAB2 SUMOylation mediated by TRIM60 (Fig. 4g). These results showed that TRIM60 functions as an E3 SUMO ligase to mediate the SUMOylation of TAB2 at K329 and K562.

### TAB2 SUMOylation inhibits the MAPK and NF-κB pathways by disrupting TRAF6/TAB2/TAK1 complex formation

**Fig. 5:**
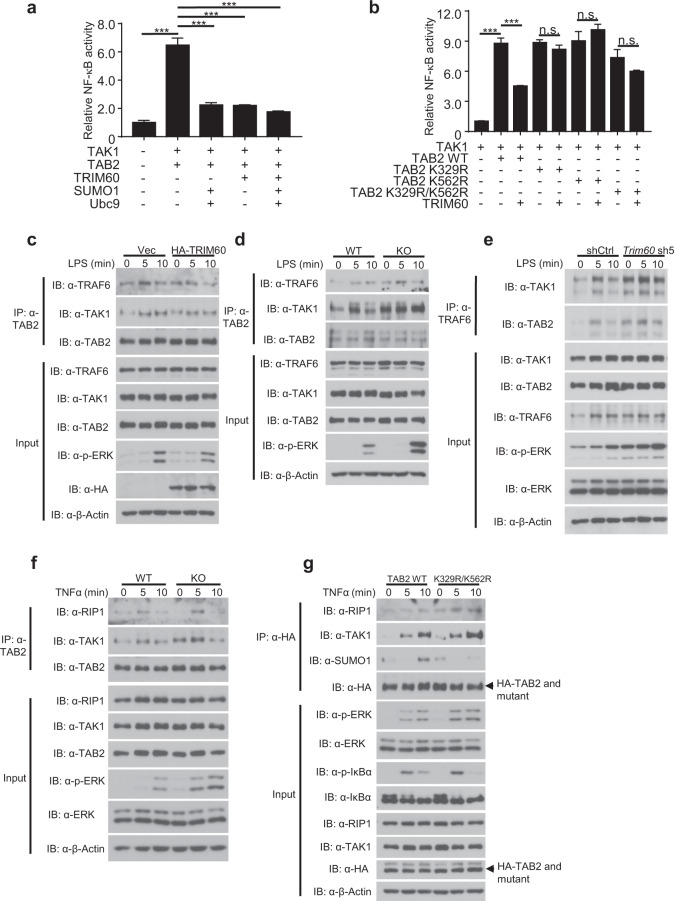
SUMOylation of TAB2 inhibits NF-κB activation by suppressing the TRAF6/TAB2/TAK1 complex. **a** Dual luciferase assay analysis of the effects of TRIM60-mediated SUMOylation on TAK1/TAB2-induced NF-κB activity. HEK293T cells were transiently transfected with the indicated plasmids, and the dual luciferase assay was performed. **b** Dual luciferase assay analysis of the effects of TAB2 mutants on the TRIM60-mediated suppression of NF-κB activity. **c** IP and WB analyses of the TRAF6/TAB2/TAK1 complex in control and HA-TRIM60-overexpressing RAW cells stimulated by LPS as indicated. Formation of the TRAF6/TAB2/TAK1 complex was examined in BMDMs (**d**) and RAW cells (**e**). Cells were stimulated with LPS for the indicated amounts of time, and IP and WB analyses were performed. **f** TRIM60 suppresses RIP1/TAB2/TAK1 signalosome formation in MEFs. IP and WB analyses of the RIP1/TAB2/TAK1 complex in MEFs. WT and TRIM60 KO MEFs were stimulated with TNFα as indicated, followed by IP and WB analyses. **g** IP and WB analyses of MAPK/NF-κB signaling activation and RIP1/TAB2/TAK1 complex formation. TAB2-deficient MEFs rescued with WT or TAB2-K329R/K562R were stimulated with TNFα as indicated, followed by IP and WB analyses to detect RIP1/TAB2/TAK1 complex formation, TAB2 SUMOylation, and phosphorylation of ERK and IκBα. The formation of the TRAF6/TAB2/TAK1 complex in **c** and **d** are quantified by ImageJ and shown as Supplementary Fig. 8a and b, respectively. The firefly luciferase activity levels in **a** and **b** were normalized to the *Renilla* luciferase activity levels and are presented as the mean ± SEM. ***P < 0.001; n.s. no significance (one-way ANOVA followed by Tukey’s multiple comparisons). The data are representative of three independent experiments (**a**–**g**)

The role of TAB2 SUMOylation in TLR signaling remains unknown. Our data thus far indicated that TRIM60 negatively regulates the TLR signaling pathway by mediating TAB2 SUMOylation; we therefore reasoned that TAB2 SUMOylation might inhibit MAPK and NF-κB activation. To examine this hypothesis, a luciferase assay was performed by cotransfecting the NF-κB reporter plasmid with TAB2 and with or without SUMO1 and Ubc9. As previously shown in Fig. 2g, overexpression of TRIM60 inhibited NF-κB activation; notably, overexpression of SUMO1 and Ubc9 also suppressed NF-κB activation (Fig. 5a). K329 and K562 were identified as the lysine residues of TAB2 targeted by TRIM60 for SUMOylation, and their roles in TRIM60-mediated suppression of NF-κB activity were further examined. As expected, overexpression of TRIM60 suppressed the NF-κB activity induced by TAB2 and TAK1; however, mutants of TAB2 harboring either a single or double mutants were largely resistant to the inhibition of NF-κB activity mediated by TRIM60 (Fig. 5b), which was consistent with the K329R/K562R mutation of TAB2 blocking TAB2 SUMOylation mediated by TRIM60 (Fig. 4g).

Since SUMOylation did not cause any appreciable TAB2 degradation (Supplementary Fig. 6c, d), we reasoned that the SUMOylation of TAB2 might interfere with the formation of the TAB2 signaling complex with TAK1 and TRAF6 or RIP1, thereby dampening downstream signaling pathways. To test this hypothesis, control and HA-TRIM60-expressing RAW cells were stimulated with LPS followed by IP with an anti-TAB2 antibody and WB analysis to detect the recruitment of TAB2 and TAK1 to TRAF6. LPS stimulation triggered the recruitment of TAB2 and TAK1 to TRAF6 (Fig. 5c); importantly, the recruitment of TAB2 and TAK1 to TRAF6 was impaired in TRIM60-expressing RAW cells (Fig. 5c and Supplementary Fig. 8a). Similarly, LPS stimulation induced the interaction of the TRAF6/TAB2/TAK1 complex (Fig. 5d). In primary BMDMs, the interaction was significantly increased in *Trim60*^*−/−*^ BMDMs (Fig. 5d and Supplementary Fig. 8b). Consistently, the recruitment of TAB2 and TAK1 to TRAF6 induced by LPS was also increased in TRIM60-knockdown RAW cells (Fig. 5e). The formation of the RIP1/TAB2/TAK1 complex was also examined in WT and *Trim60*^*−/−*^ MEFs. As indicated in Fig. 5f, the recruitment of TAB2 and TAK1 to RIP1 was increased in *Trim60*^*−/−*^ MEFs compared with WT cells. To further confirm the role of TAB2 SUMOylation in regulating TAK1 signalsome formation, a CRISPR/Cas9-mediated gene editing strategy was applied to generate TAB2-deficient MEFs, followed by reconstitution of WT or mutant TAB2 (K329R/K562R) and TNFα stimulation (Supplementary Fig. 9). As shown in Fig. 5g, TNFα stimulation-induced MAPK and NF-κB activation (indicated by phosphorylation of ERK and IκBα) and the formation of the RIP1/TAB2/TAK1 complex in MEFs. Notably, MAPK and NF-κB activation was enhanced, consistent with the increased formation of the RIP1/TAB2/TAK1 complex in MEFs reconstituted with TAB2-K329R/K562R (Fig. 5g). As expected, TAB2 SUMOylation was compromised in MEFs reconstituted with TAB2-K329R/K562R upon TNFα stimulation (Fig. 5g). These results indicated that the TRIM60-mediated SUMOylation of K329 and/or K562 of TAB2 suppressed downstream MAPK and NF-κB activation. Collectively, our data suggested that TAB2 SUMOylation mediated by TRIM60 impaired the formation of the TRAF6/TAB2/TAK1 or RIP1/TAB2/TAK1 complex and thus suppressed the activation of downstream signaling pathways.

### TRIM60-deficient mice are more susceptible to LPS-induced septic shock but resistant to *L. monocytogenes* infection

**Fig. 6:**
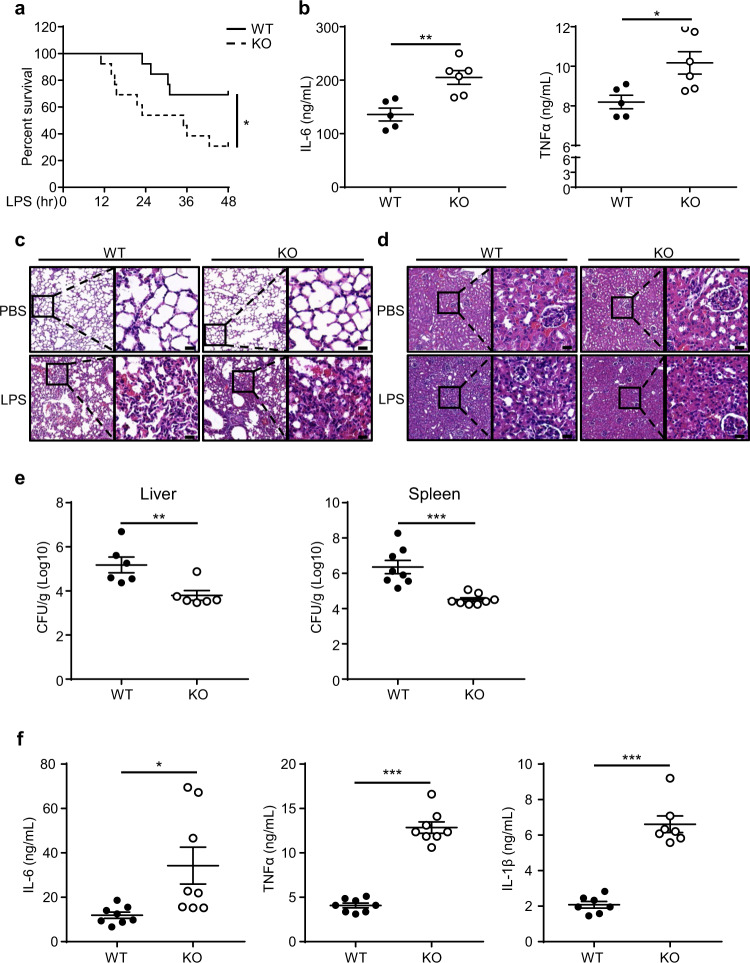
TRIM60 deficiency potentiates the TLR-mediated innate immune response in vivo. **a** Age-matched WT (*n* = 13) and *Trim60*^*−*/*−*^ (*n* = 13) mice were intraperitoneally injected (i.p.) with LPS (50 mg/kg body weight), and the survival was monitored for 48 h, followed by Kaplan–Meier analysis. **b** ELISA analysis of the IL-6 and TNFα levels in sera from WT (*n* = 5) and *Trim60*^*−*/*−*^ mice (*n* = 6) 6 h after the LPS injection. Each dot represents an individual mouse treated as indicated. H&E staining of lung (**c**) and kidney (**d**) tissues from WT and *Trim60*^*−*/*−*^ mice injected with LPS. **e, f** Six-week-old WT (*n* = 8) and *Trim60*^*−/−*^ (*n* = 8) mice were infected via the i.v. route with *L. monocytogenes* at 2 × 10^4^ CFU. The bacterial loads in the liver and spleen were examined at 72 h after infection (**e**). The proinflammatory cytokine levels in sera were tested at 24 h after infection (**f**). Each dot represents an individual mouse infected with *L. monocytogenes*. The data in **b, e,** and **f** are presented as the mean ± SEM. *P < 0.05; **P < 0.01; ***P < 0.001 (log-rank test for **a**; two-tailed unpaired Student’s *t* test for **b, e, f**). The scale bars in **c** and **d**) represent 20 μm. The results in **c** and **d** are representative of four pairs of WT and TRIM60 KO mice

To evaluate the function of TRIM60 in vivo, WT and *Trim60*^*−/−*^ mice were injected intraperitoneally with LPS to induce septic shock, and the survival was monitored. As shown in Fig. 6a, injection of LPS-induced severe septic shock in both WT and *Trim60*^*−*/*−*^ mice, as expected. TRIM60-deficient mice were more susceptible to LPS-induced shock than WT mice, with 9 out of 13 *Trim60*^*−/−*^ mice dying during the observation period of 48 h, whereas only 4 out of 13 WT mice succumbed (Fig. 6a). Sera from WT and *Trim60*^*−/−*^ mice were collected 6 h after LPS injection, and the levels of proinflammatory cytokines were measured by ELISA. In line with the survival data, TRIM60-deficient mice showed elevated serum levels of proinflammatory cytokines, such as IL-6 and TNFα (Fig. 6b). Consistently, histological examination showed that LPS injection-induced severe inflammation in the lungs and kidneys. The lung tissues of *Trim60*^*−/−*^mice showed more severer structural damage and hemorrhaging as well as more inflammatory cells infiltration than those of WT mice (Fig. 6c). In the kidneys, tissue damage was also observed, indicating more severer edema and dilation in renal tubular epithelial cells, more severer luminal narrowing of renal tubules, and more inflammatory cells infiltration in *Trim60*^*−/−*^ mice compared with WT mice (Fig. 6d). Thus, our results showed that TRIM60 protected against LPS-induced septic shock by inhibiting proinflammatory cytokine production and tissue damage.

In line with the roles of TRIM60 in the LPS-induced septic shock model, TRIM60^*−/−*^ mice were resistant to *L. monocytogenes* infection, and the bacterium preferred to replicate in the liver and spleen.^36^ WT and *Trim60*^*−/−*^ mice were intravenously (i.v.) injected with *L. monocytogenes* (2 × 10^4^ CFU), and bacterial loads in the liver and spleen were determined after 72 h. The bacterial burdens in both the livers and spleens of *Trim60*^*−/−*^ mice were significantly decreased compared with those in WT mice (Fig. 6e). Notably, this phenotype was correlated with increased serum levels of proinflammatory cytokines, such as IL-6, TNFα, and IL-1β, in *Trim60*^*−/−*^ mice (Fig. 6f). Taken together, these data demonstrated that TRIM60 played crucial roles in the immune response to LPS-induced septic shock and *L. monocytogenes* infection.

## Discussion

In this study, we explored the SUMOylation of TAB2 mediated by TRIM60 as a mechanism negatively regulating the TLR-mediated innate immune response, as it impaired the formation of the TRAF6/TAB2/TAK1 signalosome and downstream MAPK and NF-κB signaling pathways. TRIM60 deficiency in macrophages led to elevated proinflammatory cytokine production. Consistent with the in vitro results, TRIM60-deficient mice were more susceptible to LPS-induced septic shock and more resistant to *L. monocytogenes* infection than WT mice.

TAK1 is required for activation of the NF-κB and MAPK signaling pathways, as it is the kinase upstream of IKK and MAPK kinase^4^; however, the roles of TAB2 and TAB3 in TAK1 activation are arguable. Previous biochemical studies have shown that TAB2 and TAB3 are adapter proteins that activate TAK1.^37,38^ TAB2 forms a complex with TAB3 and TAK1 under steady-state conditions, which does not lead to the activation of TAK1.^39^ Upon TLR and IL-1β stimulation, TRAF6 is activated and ubiquitinated through K63-ubiquitin, thereby recruiting and activating the ubiquitin-dependent kinase TAK1 and downstream signals via the C-terminal ubiquitin-binging domain of TAB2, which is in line with structural evidence from crystallographical studies.^5,6^ Regarding physiological functions, TAB2 knockout mice die due to fetal liver degeneration,^9^ a phenotype shared by mice with deficiencies of key NF-κB components,^40^ suggesting the crucial role of TAB2 in embryo development and in the NF-κB pathway. MEFs generated from TAB2 knockout embryos showed normal MAPK and NF-κB activity upon IL-1 stimulation.^9^ Conditional TAB2 knockout mice have also been generated to study the cell-specific function of TAB2 in adult mice. Using CD19-Cre *Tab2*^*fl/fl*^, Lyz-cre *Tab2* ^*fl/fl*^ and TAB3 KO mice, Ori et al. showed that TAB2 and TAB3 are important for MAPK activation in B-cells but not in macrophages, suggesting the cell- and signaling-specific functions of TAB2 and TAB3.^11^ The notion of TAB2 redundancy in the TLR signaling pathway has also been challenged by recent reports, in which TAB2 was found to be critical for IL-1β- and RANKL-triggered signaling activation.^33,41^ The discrepancies among these data are difficult to reconcile but may be due to methodologies and the unknown complexity of TAB2 functions in different settings as well as to TAB2-dependent and TAB2-independent TAK1 activation.^42^

TAB2 is regulated at multiple levels for the fine tuning of the immune response. miR-155 regulates the IL-1 signaling pathway in human dendritic cells by directly targeting TAB2 mRNA.^16^ Additionally, the protein expression of TAB2 is modulated in a lysosome-dependent manner. TRIM38 functions as a shuttle protein to transfer TAB2 to lysosomes, independent of E3 ubiquitin ligase activity.^14^ TRIM30α might also promote TAB2/TAB3 degradation via a similar mechanism, as indicated by the colocalization of TRIM30α and TAB2 in lysosomes.^15^ Furthermore, TRIM30α also prevents the autoubiquitination of TRAF6, suggesting that TRIM30α regulates the TLR/IL-1R-triggered NF-κB and MAPK signaling pathways at different levels. However, our data suggested a novel regulatory mechanism of TAB2 by TRIM60-mediated SUMOylation. In addition to the fact that overexpression of TRIM60 did cause TAB2 degradation in HEK293T cells, which was partially blocked by NH_4_Cl treatment (the lysosome inhibitor) (Supplementary Fig. 10), we failed to detect any appreciable TAB2 degradation in primary BMDMs, RAW cells, or MEFs (Fig. 4a and Supplementary Fig. 6c, d). Instead, our data showed that TAB2 could be SUMOylated by TRIM60 upon LPS stimulation. More importantly, the SUMOylation of TAB2 was compromised in TRIM60-deficient cells (Fig. 4a, b and Supplementary Fig. 7a, b). Regarding TRIM60-mediated TAB2 SUMOylation in a RING-dependent manner in vitro, TRIM60 is proposed to be an E3 SUMO ligase for TAB2 SUMOylation.

Although no evidence indicates that SUMOylation directly causes target protein degradation, SUMOylation plays critical roles in many biological processes, including gene stability, DNA damage and repair, and the cell cycle.^43–45^ Additionally, the links between ubiquitination and SUMOylation have been studied, indicating the synergistic or competitive crosstalk of these pathways.^46,47^ For instance, the nuclear receptor NR4A1 is targeted for SUMOylation and subsequent ubiquitination by the E3 ubiquitin ligase RNF4 for degradation;^48^ another study illustrates that the E3 SUMO ligase TRIM19 and the SUMO-targeted ubiquitin ligase RNF4 synergize for protein quality control.^49^ Emerging studies also indicate that SUMOylation and ubiquitination compete to regulate protein stability. IκBα is modified by SUMOylation on lysine 21, which blocks its ubiquitination and degradation.^50,51^ Furthermore, TRIM38 mediates the SUMOylation of cyclic GMP-AMP synthase (cGAS) on K464, which suppresses the K48-associated polyubiquitination and degradation of cGAS and thus ensures an effective innate immune response to DNA viruses during the early phase of infection.^52^ In our study, the SUMOylation of TAB2 impaired the recruitment of TAB2/TAK1 to TRAF6 and further suppressed the activation of the downstream MAPK and NF-κB signaling pathways. Furthermore, we did not find any obvious alteration of TAB2 ubiquitination in the presence or absence of TRIM60 (Supplementary Fig. 6a). Collectively, these results suggest that TRIM60 mainly regulates TRAF6/TAB2/TAK1 signalosome formation by regulating TAB2 SUMOylation.

SUMOylation preferentially occurs on lysine residue(s) in the consensus motif Ψ-K-X-E.^19^ Interestingly, species ranging from zebrafish to humans exhibit two conserved TAB2 motifs (Fig. 4f), within which there are two conserved lysine residues, K329 and K562, in mouse TAB2.^53^ This strongly suggests the fundamental and critical roles of these two residues as potential sites subjected to SUMOylation. Moreover, SUMOylation of TAB2 might be a conserved mechanism to dampen TAK1 activation across species. Replacement of either lysine with arginine, or both, abolished the SUMOylation of TAB2 by TRIM60, as indicated by an in vitro SUMOylation assay (Fig. 4g), and thereby promoted TNFα-induced RIP1/TAB2/TAK1 complex formation and downstream MAPK/NF-κB activation (Fig. 5g). Consistently, TAB2 harboring these mutations was resistant to the TRIM60-mediated suppression of NF-κB activity, as indicated by the luciferase assay results (Fig. 5b). A crystallographic study indicated that the ZF domain of TAB2 is critical for its interaction with K63 polyubiquitin chains^5,6^ but is not required for the TRIM60-TAB2 interaction (Fig. 3e). It is plausible that TRIM60-mediated TAB2 SUMOylation might interfere with the interaction of TAB2 with K63 polyubiquitin chains for signalosome formation, but this hypothesis remains hypothetical. Taken together, our data reveal a novel modification of TAB2 mediated by TRIM60 and provide genetic and biochemical evidence that TRIM60-mediated TAB2 SUMOylation serves as a negative regulatory mechanism of the TLR signaling pathway and the innate immune response.

## Methods and materials

### Animals

TRIM60 knockout mice were generated by crossing *Trim60*^*f/f*^ mice with Ella-Cre mice on the C57BL/6 background. All animals were maintained in a specific pathogen-free (SPF) facility at the State Key Laboratory of Biotherapy, Sichuan University, China. Animal use and care were approved by the State Key Laboratory of Biotherapy, Sichuan University in accordance with the Institutional Animal Care and Use Committee Guidelines.

### Plasmids

Hemagglutinin (HA)- and Myc-tagged TRIM60 and Myc-tagged TAB2 were cloned into the pCDH-copGFP lentiviral vector or pCMV vector. Flag-tagged TAB2 was cloned into the pCMV6 vector. Different truncated mutations of TRIM60 and TAB2 were constructed using standard molecular cloning techniques. TAB2 mutants (K329R, K562R, K329R/K562R) were generated using a QuickChange Site-Directed Mutagenesis kit (Agilent), with pCMV-HA-TAB2 as a template. The cDNA of Ubc9 was synthesized by GENEWIZ and cloned into the pCMV6 vector. For bacterial expression, full-length or truncated TRIM60, TAB2 and TAB2 mutants were constructed in the pGST2 plasmid. The pRK-His-SUMO1 and pX458 plasmids were kind gifts from Dr. Hongbing Shu (Wuhan University) and Dr. Peng Lei (Sichuan University), respectively. All plasmids were confirmed by sequencing.

### Antibodies and reagents

Antibodies against phospho-ERK1/2 (4370), phospho-p38 (9215), phospho-JNK1/2 (9251), phospho-IκBα (9246), TAK1 (4505), TAB2 (Leu 330, 3745), SUMO1 (4930), and HA-tag (3724) were obtained from Cell Signaling Technology. Antibodies against ERK1/2 (201245-4A4), p38 (200782), JNK1/2 (201001), IκBα (200517) and TRAF6 (380803) were obtained from Zen Bioscience, China. Anti-β-Actin (A2228), anti-Flag (F1804), and HRP-conjugated anti-Flag (A8592) antibodies were purchased from Sigma-Aldrich. Anti-Myc (MA1-21316), HRP-conjugated anti-Myc (MA1-81357), and HRP-conjugated goat anti-rabbit IgG (31460) antibodies were purchased from Invitrogen. Antibodies against TRAF6 (SC-8409), TAB2 (N48-73, SC-398188), and GST (SC-53909) were obtained from Santa Cruz Biotechnology. The antibodies against RIP1 (610458), ubiquitin (04-263), and K63-linked ubiquitin (BML-PW0600) were purchased from BD Bioscience, Merck Millipore, and Enzo Life Science, respectively. The HRP-conjugated anti-mouse secondary antibody (KCB002) was purchased from Rockland.

LPS (*E. coli* O127:B8, L3129), MG132 (474790), polybrene (H9268) and chloroquine (C6628) were purchased from Sigma-Aldrich. CpG-ODN 1826 (tlrl-1826) and puromycin (540411) were obtained from InvivoGen and Merck Millipore, respectively. Murine TNFα (AF-315-01A) was purchased from PeproTech.

### Cell culture and stimulation

BMDMs were cultured as described previously.^54^ Briefly, the bone marrow cells were flushed from the femurs and tibias of adult mice (6–8 weeks old) and cultured in Dulbecco’s modified Eagle’s medium (DMEM) supplemented with 20% fetal bovine serum (FBS) and 30% L929 conditional medium for 7 days to induce differentiation into macrophages. For stimulation, macrophages were plated into 12- or 6-well plates and stimulated with different stimuli, including LPS (20 ng/mL) and CpG-ODN 1826 (2 μM), for the indicated time periods.

To prepare MEFs, breeding pairs were established using heterozygous male and female mice. On days 12.5–14.5, embryos were used to prepare MEFs and cultured in DMEM supplemented with 10% FBS. For TNFα stimulation, MEFs were plated in 6-well plates and stimulated with TNFα (20 ng/mL) for the indicated time periods.

HEK293T and RAW cells were obtained from ATCC and maintained in DMEM containing 10% FBS. TRIM60-knockdown RAW cells and *Trim60*^*−*/*−*^ MEFs reconstituted with TRIM60 and their parental cell lines were generated through lentiviral infection. All the cell lines were free of mycoplasma contamination.

### IP-mass spectrum analysis

RAW cells overexpressing HA-TRIM60 were stimulated with LPS for 30 min. TRIM60-interacting proteins were purified by immunoprecipitation using anti-HA magnetic beads (88836, Pierce). The IP products were subjected to mass spectrometric analysis. Briefly, the washed IP beads were boiled in 30 μl of 1X NUPAGE SDS sample buffer (Invitrogen) and then separated by SDS-PAGE (NuPAGE 10% Bis-Tris Gel, Invitrogen). The proteins in the gel were visualized by Coomassie Brilliant blue staining and divided into several gel pieces according to molecular weight. The individual gel pieces were destained, and proteins were subjected to in-gel digestion with trypsin (Promega). The tryptic peptides were eluted, lyophilized, resuspended in 10 μl of loading solution (5% methanol containing 0.1% formic acid) and analyzed by nano-liquid chromatography-tandem mass spectrometry (LC-MS/MS) with a nano-LC 1000 system (Thermo Fisher Scientific) coupled to a Q Exactive plus (Thermo Fisher Scientific) mass spectrometer. The raw MS data were analyzed by Mascot.

### Immunoprecipitation and western blot analysis

Cell lysates were prepared and subjected to immunoprecipitation and immunoblot assays as described previously.^55^ In brief, cells were collected and lysed with RIPA buffer containing a complete protease inhibitor cocktail (Roche). Total cell lysates were centrifuged for 10 min at 13000 rpm and 4 °C, and the supernatants were incubated with an IP antibody at 4 °C overnight and then with protein A/G beads (P2028, Beyotime Biotechnology) for 1 h. The beads were washed five times with RIPA buffer, and the immunoprecipitants were eluted from the beads by boiling for 5 min with 2 × SDS protein loading buffer and then subjected to western blot analysis.

To detect the endogenous interaction of TAB2 with TRIM60, control and HA-TRIM60-overexpressing RAW cells stimulated with LPS (20 ng/mL) for 10 min were lysed with NP-40 lysis buffer containing protease inhibitors and subjected to immunoprecipitation with anti-HA magnetic beads (88836, Pierce) according to the manufacturer’s instructions. Proteins were separated by SDS-PAGE and analyzed by western blot with the indicated antibodies.

### Endogenous SUMOylation assay

To detect endogenous TAB2 SUMOylation, the indicated cells were lysed in RIPA buffer containing complete protease inhibitors plus 1% SDS and denatured by boiling for 5 min. After dilution of the lysates with regular RIPA buffer 10 times and sonication for 2 min, the supernatants were subjected to immunoprecipitation with an anti-TAB2 antibody. The precipitated proteins were then subjected to western blot analysis with the SUMO1 antibody.

### Protein purification and in vitro SUMOylation assay

GST-tagged TRIM60, TRIM60 ΔRING, TAB2, and TAB2 mutants were expressed in a modified *E. coli* BL21 strain stably expressing GroEL and GroES at 16 °C overnight. Cells were harvested and resuspended in buffer (25 mM Tris (pH = 8.0), 150 mM NaCl) containing 1 mM PMSF, 2 mM DTT and 1 mM ATP, followed by homogenization via French Press and centrifugation to obtain the supernatant. The proteins were purified by an affinity technique using GS4B agarose beads (QIAGEN), elution with buffer (50 mM Tris (pH = 8.0), 150 mM NaCl, 2 mM DTT) containing 10 mM reduced glutathione (GSH), and subjection to a Superdex 200 (GE Healthcare) or Superose 6 (GE Healthcare) chromatography column on an ACTA system (GE Healthcare). Purified proteins were identified by SDS-PAGE.

In vitro SUMOylation assays were performed using a SUMOlink SUMO-1 kit (40120, Active Motif) according to the manufacturer’s instructions. In brief, GST-tagged TAB2 (0.3 μg) was incubated with either the GST-TRIM60 (0.6 μg) or GST-TRIM60 ΔRING protein in the presence of E1 activating enzyme, E2 conjugating enzyme and the SUMO1 protein at 30 °C for 3 h. The reaction was terminated by an equal volume of 2 × SDS protein loading buffer and boiling at 90 °C for 3 min; the proteins were then subjected to western blot analysis with an anti-SUMO1 antibody.

### Lentivirus package and infection

To generate stable TRIM60-knockdown RAW cells, HEK293T cells were cotransfected with pLKO.1 lentiviral vectors harboring *Trim60*-shRNA together with psPAX2 and pMD2. G packaging plasmids. The medium was replaced with fresh complete medium 8 h after transfection, and the supernatants containing lentiviral particles were collected 48 h after transfection. RAW cells were infected with the lentiviral particles for 8 h in the presence of polybrene (8 μg/mL). Two days after infection, cells were selected in medium containing puromycin (3 μg/mL).

For the reconstitution of TRIM60 into *Trim60*^*−/−*^ MEFs, cells were infected with a lentiviral vector (pCDH-copGFP-HA-TRIM60) in the presence of polybrene (8 μg/mL) for 8 h. After 48 h, GFP-positive cells were sorted by FACS (Aria III, BD Bioscience).

### Generation of TAB2-deficient MEFs

To generate TAB2-deficient MEFs, two single-guide RNAs (sgRNA) targeting exon 3 of *Tab 2*, sgRNA-3 (5’-GTGATGGACAGCTTCACGGT-3’) and sgRNA-4 (5’- GCACCAGCTCAAGTTCCTCA-3’), were designed, ligated into the pX458 plasmid and transfected into MEFs by electroporation on a 4D-Nucleofector™ X Unit (Lonza) according to the manufacturer’s instructions.^56^ Twenty-four hours after transfection, GFP-positive cells were sorted by FACS and then seeded into 96-well plates by the limited dilution method.^57^ Single-cell colonies were expanded for ~2 weeks, and genomic DNA was extracted from each single colony. The genomic region flanking the gRNA target site was amplified by PCR using rTaq polymerase (TaKaRa, RR901) and inserted into the pMD19 T-Vector (TaKaRa, 3271) for sequencing. WB was used to further confirm the deficiency of TAB2. To rescue TAB2 expression in TAB2-knockout MEFs, HA-TAB2 WT or HA-TAB2 K329R/K562R was induced into TAB2-knockout MEFs by lentiviral infection and confirmed by WB.

### Real-time PCR and ELISA

Total RNA was extracted from the indicated cells using TRIzol reagent (Invitrogen) and reverse transcribed into cDNA with an iScript cDNA Synthesis Kit (Bio-Rad). Relative gene expression was then analyzed by qPCR using 2 × iTaq Universal SYBR Green Supermix (Bio-Rad) on a CFX Connect Real-Time System (Bio-Rad). The primers used in this study are listed in Supplementary Table 1.

The enzyme-linked immunosorbent assay (ELISA) was performed using mouse IL-6, TNFα, and IL-1β ELISA kits (eBioscience) according to the manufacturers’ instructions.

### Luciferase assay

HEK293T cells were plated on 24-well plates and transfected with the indicated plasmids together with the NF-κB and pRL-TK *Renilla* luciferase reporter plasmids using Lipofectamine 2000 (Invitrogen) according to the standard protocol. When necessary, the empty control plasmid was added to ensure equal amounts of total DNA were used for each transfection. For the luciferase assay in HEK293T cells, NH_4_Cl was added at a final concentration of 25 mM at 6 h before cell harvest. Forty-eight hours post transfection, the cells were harvested, and relative firefly luciferase activity was determined using the Dual Luciferase Reporter Assay kit (Promega) according to the manufacturer’s instructions. Relative firefly luciferase activity was normalized to that of *Renilla* luciferase.

For the luciferase assay in MEFs, cells were transiently transfected with the NF-κB and pRL-TK *Renilla* luciferase reporter plasmids through electroporation on a 4D-Nucleofector™ X Unit (Lonza). After 24 h, the cells were stimulated with TNFα (20 ng/mL) for an additional 6 h and then subjected to a dual luciferase reporter assay to determine relative firefly luciferase activity.

### LPS-induced septic shock

Six- to eight-week-old male WT and *Trim60*^*−/−*^ mice were injected with LPS (O127:B8, 50 mg/kg body weight) intraperitoneally. The mice were monitored for survival for 48 h. Serum was collected at 6 h after LPS injection for ELISA analysis, and organs (lung and kidney) were collected at 24 h after LPS injection for hematoxylin and eosin (H&E) staining to examine tissue damage.

### L. monocytogenes culture and in vivo infection

*L. monocytogenes* was kindly provided by Dr. Jiyan Zhang, Institute of Basic Medical Sciences, China and cultured in brain heart infusion (BHI) medium containing erythromycin (5 μg/mL) at 37 °C overnight on a shaking platform. For in vivo infection, bacteria were diluted in cold PBS to 10^5^ CFU/mL and injected intravenously in a volume of 200 μL (2 × 10^4^ CFU). Twenty-four hours after injection, blood samples were collected for proinflammatory cytokine detection by ELISA. Bacterial loads in the liver and spleen were examined at 72 h after injection. Briefly, the livers and spleens of infected mice were homogenized in 1 mL of cold PBS containing 0.5% Triton X-100, serially diluted in BHI medium and plated on BHI agar containing erythromycin (5 μg/mL). Bacterial CFUs were cultured after the plates were incubated for 24 h at 37 °C.^58^

### Statistical analysis

Statistical analysis was performed using Prism software. One- or two-way ANOVA followed by Tukey’s multiple comparisons test was used to compare differences between multiple groups. Two-tailed unpaired Student’s *t* test was used to compare differences between two groups. The log-rank test was used for Kaplan–Meier analysis of survival. A *P* value less than 0.05 was considered statistically significant. The levels of significance are indicated as *P < 0.05; **P < 0.01; ***P < 0.001; and n.s. no significance (*P* > 0.05).

## Supplementary information

Supp Figure 1-10

Supp table

Supplementary figure legends
