# Supplementary material for: The SUMOylation of TAB2 mediated by TRIM60 inhibits MAPK/NF-κB activation and the innate immune response
Source: Cell Mol Immunol. 2020 Nov 12;18(8):1981–94. doi: 10.1038/s41423-020-00564-w (PMC8322076; doi:10.1038/s41423-020-00564-w)

Supplementary Fig. 1

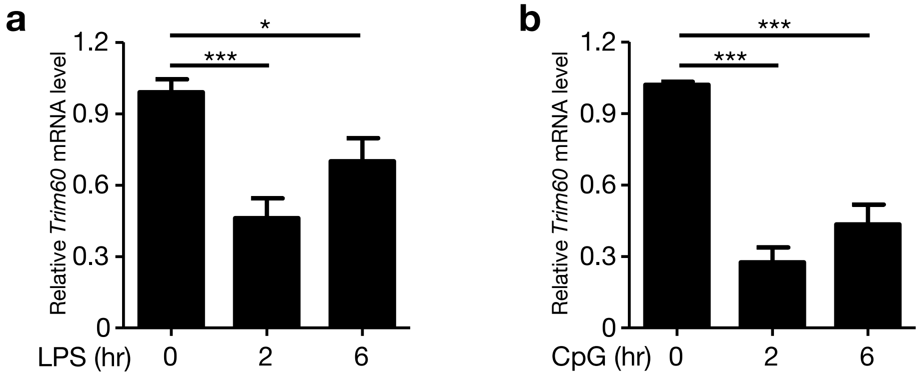

Supplementary Fig. 2

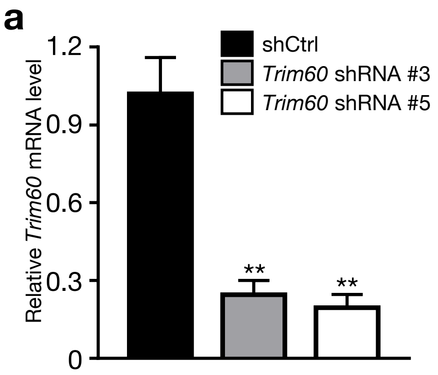

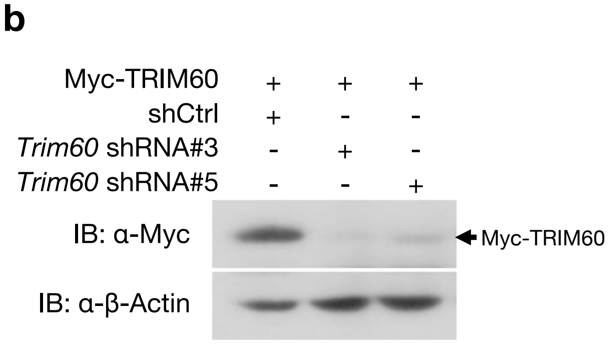

Supplementary Fig. 3

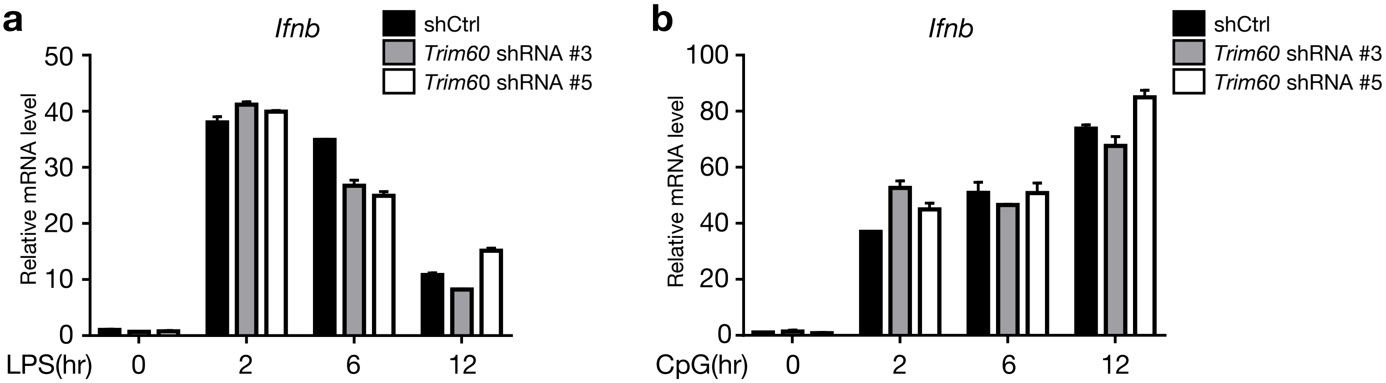

Supplementary Fig. 4

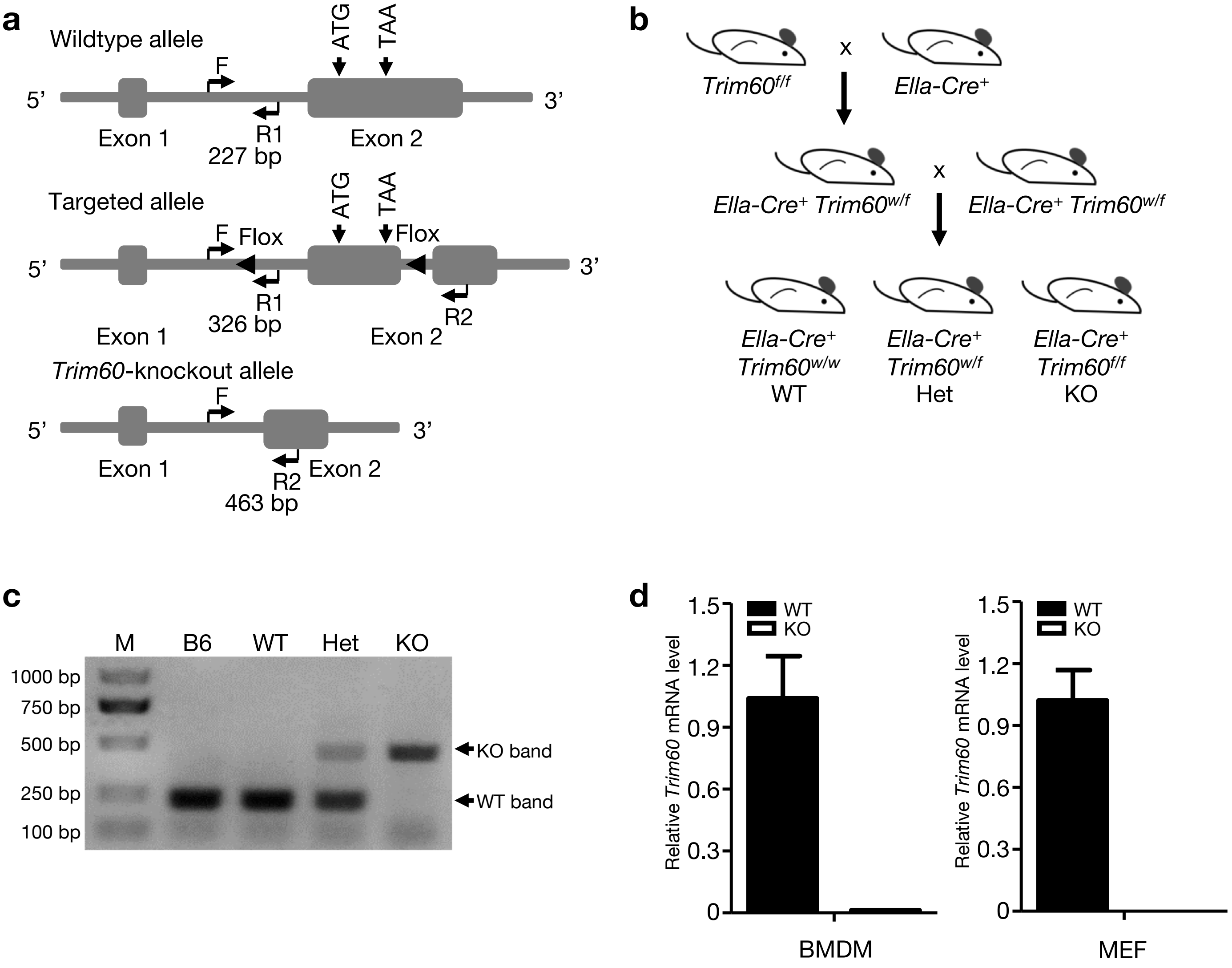

Supplementary Fig. 5

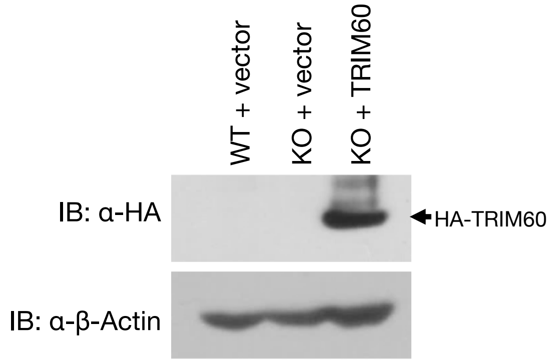

Supplementary Fig. 6

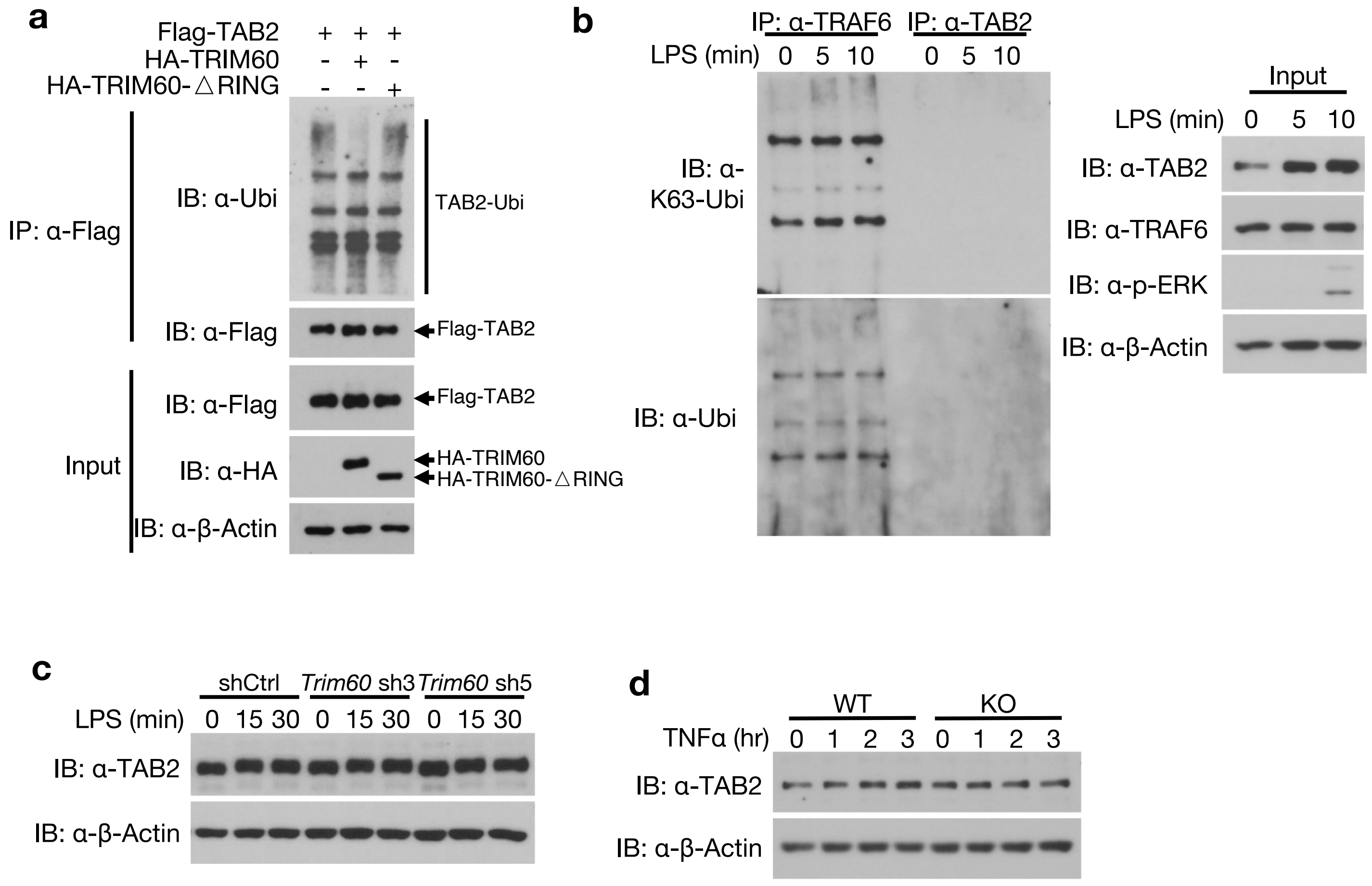

Supplementary Fig. 7

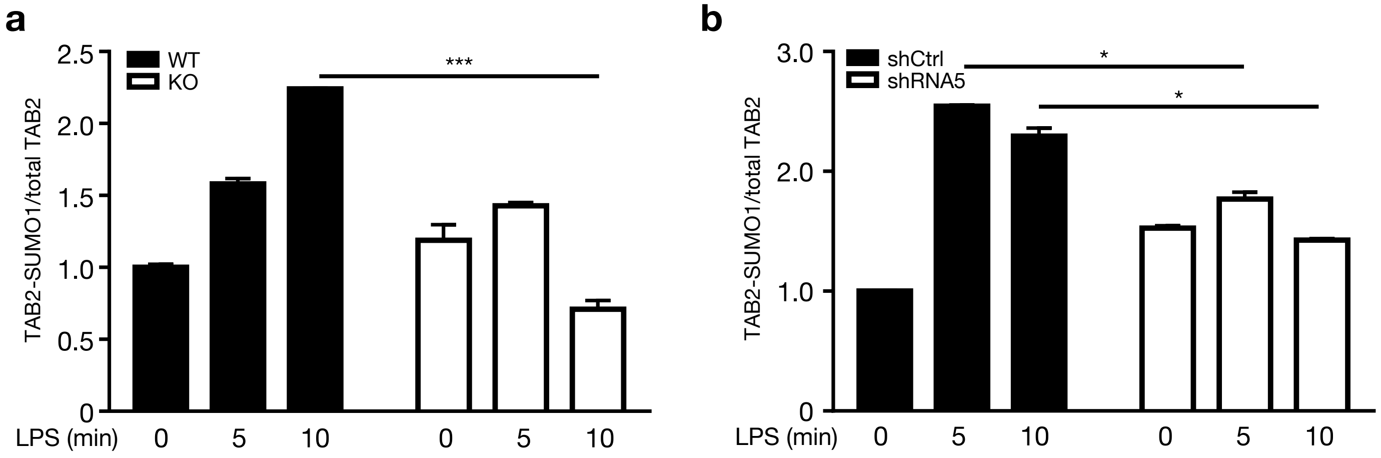

Supplementary Fig. 8

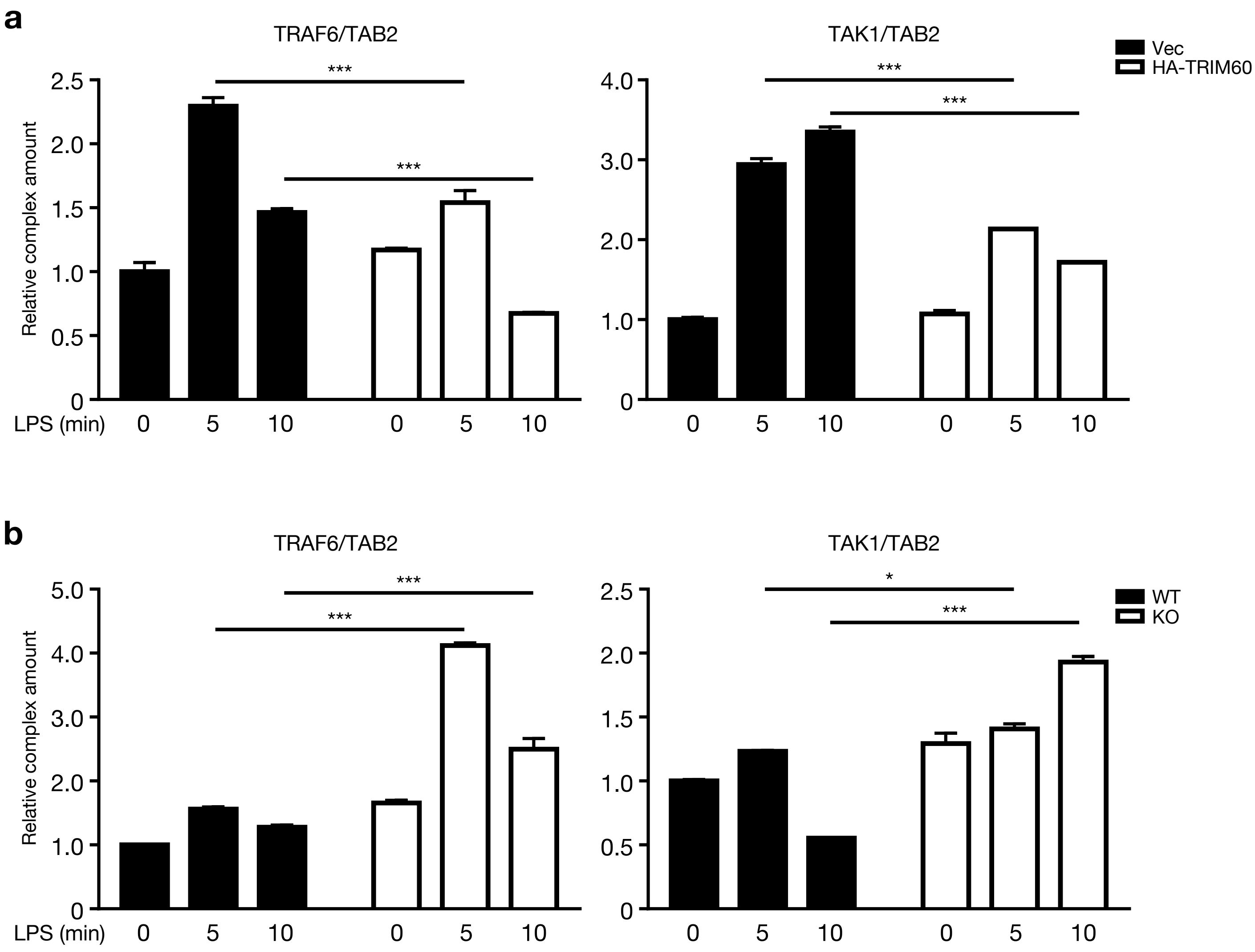

Supplementary Fig. 9

a

Tab2 WT

5’-AGACAGCACCAGCTCAAGTTCCTCAAGGCTTTAATGTTTTT----CATCTTGGATTTCACCTAGGC-3’

380 390 400 410 420 460 470 480

5’-AGACAGCACCAGCTCAAGTTC - TCAAGGCTTTAATGTTTTT----CATCTTGGATTTCACCTAGGC-3’

Tab2 KO_ gRNA4 #2 allele 1

Stop

Tab2 WT

5’-AGACAGCACCAGCTCAAGTTCCTCAAGGCTTTAATGTTTTT----CATCTTGGATTTCACCTAGGC-3’

380 390 400 410 420 460 470 480

5’-AGACAGCACCAGCTCAAGTTCC - CAAGGCTTTAATGTTTTT----CATCTTGGATTTCACCTAGGC-3’

Tab2 KO_ gRNA4 #2 allele 2

Stop

b

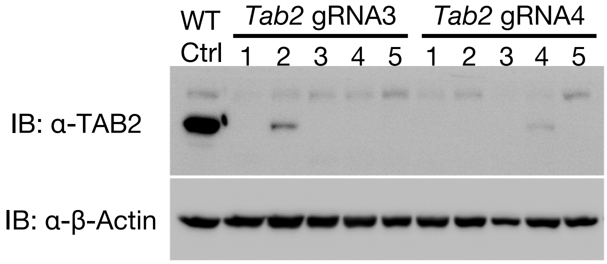

c

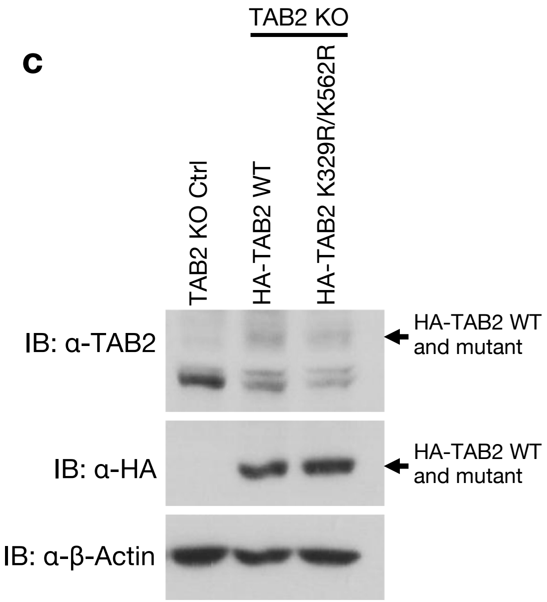

Supplementary Fig. 10

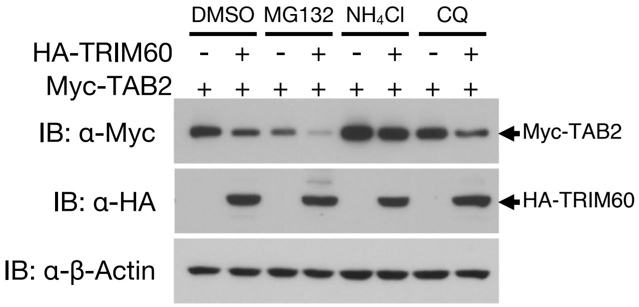

Supplement: Supplementary file 1 — Supp Figure 1-10 [file 41423_2020_564_MOESM1_ESM.pdf]
