# Supplementary material for: The SUMOylation of TAB2 mediated by TRIM60 inhibits MAPK/NF-κB activation and the innate immune response
Source: Cell Mol Immunol. 2020 Nov 12;18(8):1981–94. doi: 10.1038/s41423-020-00564-w (PMC8322076; doi:10.1038/s41423-020-00564-w)
Supplement: Supplementary file 2 — Supp table [file 41423_2020_564_MOESM2_ESM.pdf]

Supplementary table

Table 1 Primers used for real-time PCR in this study

Trim60 forward	5’-AGCACAGAAGGGTTCTTGGA-3’
Trim60 reverse	5’-CCTCCATGTGGCTCTCATTT-3’
Il6 forward	5’-TGAACAACGATGATGCACTTGC-3’
Il6 reverse	5’-GCTATGGTACTCCAGAAGACC-3’
Tnfa forward	5’-CATCTTCTCAAAATTCGAGTGACAA-3’
Tnfa reverse	5’-CCAGCTGCTCCTCCACTTG-3’
Il12b forward	5’-CCATTGAACTGGCGTTGGAAG-3’
Il12b reverse	5-ACTTGAGGGAGAAGTAGGAATGG-3’
Nos2 forward	5’-CACCTTGGAGTTCACCCAGT-3’
Nos2 reverse	5’-ACCACTCGTACTTGGGATGC-3’
Il1b forward	5’-CAACCAACAAGTGATATTCTCCATG-3’
Il1b reverse	5’-GATCCACACTCTCCAGCTGCA-3’
Ifnb forward	5’-AGCTCCAAGAAAGGACGAACAT-3’
Ifnb reverse	5’-GCCCTGTAGGTGAGGTTGATCT-3’
Ccl2 forward	5’-CCCTGTCATGCTTCTGG-3’
Ccl2 reverse	5’-TCATTGGGATCATCTTGC-3’
Ccl5 forward	5’-CCCTCACCATCATCCTCACT-3’
Ccl5 reverse	5’-CCTTCGAGTGACAAACACGA-3’
18S rRNA forward	5’-CTTAGAGGGACAAGTGGCG-3’
18S rRNA reverse	5’-ACGCTGAGCCAGTCAGTGTA-3’
Rpl13a forward	5’-AGTATCTGGCCTTTCTCCGG-3’
Rpl13a reverse	5’-CCGAACAACCTTGAGAGCAG-3’
