# Supplementary material for: The SUMOylation of TAB2 mediated by TRIM60 inhibits MAPK/NF-κB activation and the innate immune response
Source: Cell Mol Immunol. 2020 Nov 12;18(8):1981–94. doi: 10.1038/s41423-020-00564-w (PMC8322076; doi:10.1038/s41423-020-00564-w)
Supplement: Supplementary file 3 — Supplementary figure legends [file 41423_2020_564_MOESM3_ESM.docx]

**Supplementary information**

**Supplementary figure legends**

**Supplementary Fig. 1 Downregulation of *Trim60* expression upon TLR stimulation.** Wild-type BMDMs were stimulated with either LPS (**a**) or CpG (**b**) for the indicated time periods and then subjected to qPCR analysis to determine *Trim60* mRNA expression. The data in (**a, b**) are presented as the mean ± SEM of four biological replicates. *, *P* < 0.05; ***, *P* < 0.001 (one-way ANOVA followed by Tukey’s multiple comparisons).

**Supplementary Fig. 2 Knockdown of TRIM60 by shRNA.** (**a**) qPCR analysis of *Trim60* knockdown efficiency. RAW cells were infected with lentiviral particles encoding either control shRNA (shCtrl) or *Trim60*-specific shRNA (*Trim60* shRNA #3 or *Trim60* shRNA #5) and selected with puromycin. *Trim60* mRNA expression was detected by qPCR, and *Rpl13a* served as the reference gene. (**b**) HEK293T cells were transfected with Myc-TRIM60 together with either a control shRNA or *Trim60*-specific shRNA (shRNA #3 or shRNA #5). TRIM60 knockdown efficiency was determined by WB. The data in (**a**) are shown as the mean ± SEM. **, *P* < 0.01 (one-way ANOVA followed by Tukey’s multiple comparisons). The data are representative of three independent experiments (**a, b**).

**Supplementary Fig. 3 TRIM60 knockdown does not affect LPS- and CpG-induced *Ifnb* expression in macrophages.** (**a, b**) qPCR analysis of *Ifnb* expression in control (shCtrl) and TRIM60-knockdown (*Trim60* shRNA #3 and *Trim60* shRNA #5) RAW cells stimulated with either LPS (**a**) or CpG (**b**) as indicated; *18S rRNA* served as the reference gene. The data are shown as the mean ± SEM and are representative of three independent experiments (**a, b**).

**Supplementary Fig. 4 Generation of TRIM60-knockout mice.** (**a**) Overview of the *Trim60*-targeting strategy. Two *Flox* sequences were inserted upstream and inside of the 2nd exon of the *Trim60* gene, which is the protein-coding region. In Ella-Cre^+^ *Trim60^f/f^* mice, the *Trim60* protein-coding region was removed, resulting in a 463-bp product as a knockout allele when primers F and R2 were used for genotyping, while the WT (Ella-Cre^+^ *Trim60^w/w^*) mice produced a 227-bp band when primers F and R1 were used for genotyping. (**b**) Schematics of the cross strategy used to generate TRIM60 knockout mice. (**c**) Genotyping results of Ella-Cre^+^ *Trim60^w/w^,* Ella-Cre^+^ *Trim60^w/f^*, Ella-Cre^+^ *Trim60^f/f^* mice. Genomic DNA was isolated and used as a template for PCR amplification. M, molecular marker; B6, C57BL/6 control; WT, wild-type, Ella-Cre^+^ *Trim60^w/w^*; Het, heterozygous, Ella-Cre^+^ *Trim60^w/f^*; KO, knockout, Ella-Cre^+^ *Trim60^f/f^*. (**d**) qPCR analysis to validate *Trim60* deficiency in BMDMs (*left*) and MEFs (*right*); *Rpl13a* served as the reference gene. The data in (**d**) are presented as the mean ± SEM and are representative of three independent experiments.

**Supplementary Fig. 5 TRIM60 reconstitution in *Trim60^-/-^* MEFs.** WT and *Trim60*^-/-^MEFs were infected with either an empty vector or HA-TRIM60-encoding lentiviral particles. The expression of exogenous TRIM60 was determined by WB. The data are representative of three independent experiments.

**Supplementary Fig. 6 TRIM60 is dispensable for the ubiquitination and degradation of TAB2.** (**a**) TRIM60 did not affect TAB2 ubiquitination. HEK293T cells were transfected with the indicated plasmids. HEK293T cells were transiently transfected with the indicated plasmids and TAB2 ubiquitination was detected by IP and WB. (**b**) RAW cells were stimulated with LPS as indicated; ubiquitination of TAB2 and TRAF6 was determined by IP and WB. Data are representative of three independent experiments (**a, b**). (**c**) Control and TRIM60-knockdown RAW cells were stimulated with LPS for the indicated amounts of time. WB was used to detect TAB2 protein levels; β-Actin served as the loading control. (**d**) Wild-type (WT) and TRIM60-knockout (KO) MEFs were stimulated with TNFα as indicated, and WB analysis was performed to assess TAB2 protein levels; β-Actin was used as the loading control. The data are representative of at least three independent experiments (**c, d**).

**Supplementary Fig. 7 TRIM60 regulates TAB2 SUMOylation.** (**a, b**) Quantification of TAB2 SUMOylation (SUMOylated TAB2 vs total TAB2) was performed by ImageJ, and the data are presented as the mean ± SEM. *, *P* < 0.05; ***, *P* < 0.001 (two-way ANOVA followed by Tukey’s multiple comparisons). The data in (**a, b**) are related to the results in Fig. 4a, and b, respectively.

**Supplementary Fig. 8 TRIM60 suppresses TRAF6/TAB2/TAK1 signalosome formation in macrophages.** (**a, b**) Relative densitometric diagrams of TRAF6/TAB2/TAK1 complex formation in macrophages. Control and HA-TRIM60-overexpressing RAW cells (**a**) or WT and TRIM60 KO BMDMs (**b**) were stimulated by LPS as indicated, and IP and WB were performed to examine TRAF6/TAB2/TAK1 complex formation. The relative densitometric ratios of TRAF6/TAB2/TAK1 complex amounts were calculated by TRAF6/TAB2 and TAK1/TAB2 with ImageJ software and are presented as the mean ± SEM. *, *P* < 0.05; ***, *P* < 0.001 (two-way ANOVA followed by Tukey’s multiple comparisons). The data in (**a, b**) are related to the results in Fig. 5c, and d, respectively.

**Supplementary Fig. 9 Generation of TAB2-deficient MEFs by the CRISPR-Cas9 system.** (**a**) gRNA4 was used to generate TAB2-deficient MEFs by causing premature stop codons. The site targeted by gRNA4 is underlined, and the premature stop codon is indicated. (**b**) TAB2 knockout was confirmed by WB. (**c**) Reconstituted TAB2 (WT or TAB2 K329R/K562R) in TAB2-deficient MEFs was examined by WB.

**Supplementary Fig. 10 TRIM60 regulates TAB2 stability in the overexpression system.** HEK293T cells were transfected with the indicated plasmids. Thirty-six hours after transfection, the cells were treated with MG132 (20 μM, proteasome inhibitor), NH_4_Cl (25 mM, lysosome inhibitor), or chloroquine (CQ, 50 μM, autophagolysosome inhibitor) for an additional 6 h, and WB was performed to determine TAB2 protein levels; β-Actin served as the loading control. The data are representative of three independent experiments.
